# Differential Cell Sensitivity between OTA and LPS upon Releasing TNF-α

**DOI:** 10.3390/toxins2061279

**Published:** 2010-06-01

**Authors:** Lauy Al-Anati, Ebtisam Essid, Ulla Stenius, Knut Beuerlein, Klaus Schuh, Ernst Petzinger

**Affiliations:** 1 Institute of Pharmacology and Toxicology, College of Veterinary Medicine, Justus Liebig University Giessen. Frankfurter Street 107, D-35392 Giessen, Germany; Email: lauy.al-anati@ki.se (L.A.-A.); 2 Institute of Environmental Medicine, Karolinska Institutet, S-17177 Stockholm, Sweden; 3 Rudolf-Buchheim-Institute of Pharmacology, College of Medicine, Justus Liebig University Giessen, Frankfurter Street 107, D-35392 Giessen, Germany

**Keywords:** ochratoxin A, lipopolysaccharide, tumor necrosis factor α, Kupffer cells, macrophages, rat liver sinusoidal endothelial cells, HepG2 cells, rat hepatocytes

## Abstract

The release of tumor necrosis factor α (TNF-α) by ochratoxin A (OTA) was studied in various macrophage and non-macrophage cell lines and compared with *E. coli* lipopolysaccharide (LPS) as a standard TNF-α release agent. Cells were exposed either to 0, 2.5 or 12.5 µmol/L OTA, or to 0.1 µg/mL LPS, for up to 24 h. OTA at 2.5 µmol/L and LPS at 0.1 µg/mL were not toxic to the tested cells as indicated by viability markers. TNF-α was detected in the incubated cell medium of rat Kupffer cells, peritoneal rat macrophages, and the mouse monocyte macrophage cell line J774A.1: TNF-α concentrations were 1,000 pg/mL, 1,560 pg/mL, and 650 pg/mL, respectively, for 2.5 µmol/L OTA exposure and 3,000 pg/mL, 2,600 pg/mL, and 2,115 pg/mL, respectively, for LPS exposure. Rat liver sinusoidal endothelial cells, rat hepatocytes, human HepG2 cells, and mouse L929 cells lacked any cytokine response to OTA, but showed a significant release of TNF-α after LPS exposure, with the exception of HepG2 cells. In non-responsive cell lines, OTA lacked both any activation of NF-κB or the translocation of activated NF-κB to the cell nucleus, *i.e.*, in mouse L929 cells. In J774A.1 cells, OTA mediated TNF-α release via the pRaf/MEK 1/2-NF-κB and p38-NF-κB pathways, whereas LPS used pRaf/MEK 1/2–NF-κB, but not p38-NF-κB pathways. In contrast, in L929 cells, LPS used other pathways to activate NF-κB. Our data indicate that only macrophages and macrophage derived cells respond to OTA and are considered as sources for TNF-α release upon OTA exposure.

## Abbreviations

OTA: ochratoxin A
TNF: tumor necrosis factor
LPS: lipopolysaccharide
NF-κB: nuclear factor kappa B

## 1. Introduction

Ochratoxin A (OTA) is a natural mycotoxin produced from *Aspergillus* and *Penicillium* species. OTA causes diverse toxicological responses: it is genotoxic, carcinogenic, nephrotoxic, hepatotoxic, embryotoxic, teratogenic, and immunotoxic. Previous risk assessments have studied its genotoxicity and OTA-mediated nephrotoxicity [1,2,3,4]. It is also a strong immunosuppressant fungal compound [5]. The immune system has been considered as sensitive as the kidney though, because it was concomitantly affected by the lowest dose causing nephrotoxicity [6]. In addition to a reduction in size of vital immune organs, OTA immunotoxicity includes hypocellularity of bone marrow and impairment of immunoglobin responses [5]. Furthermore, its toxicity on rat livers triggers the release of inflammatory cytokines [7]. A marked release of TNF-α and IL-6 has been observed during perfusion of the isolated rat liver with OTA via the portal vein [8]. This rat liver-derived TNF-α originated from Kupffer cells [9]. Its release was affected by eicosanoids, whereby leucotrienes and CYP-450 metabolites stimulated cytokine release, whereas arachidonic acid and cyclooxygenase-derived metabolites suppressed it [7].

In blood-free perfused rat livers, a synergistic effect on OTA-mediated TNF-α release by co-addition of low doses of *E. coli* lipopolysaccharides (LPS) has been observed [10] Thus, the question arises whether in general OTA mimics LPS-induced TNF-α release in all LPS-sensitive macrophage and non-macrophage cells, indicating a release pathway similar to the LPS-triggered cascade [11,12] Therefore, in this study we compared the ability of OTA to release TNF-α from macrophage and non-macrophage cells with LPS, and investigated the possible mediator mechanismof this release.

## 2. Results and Discussion

### 2.1. Cytotoxicity Effects of OTA and LPS

**Figure 1:**
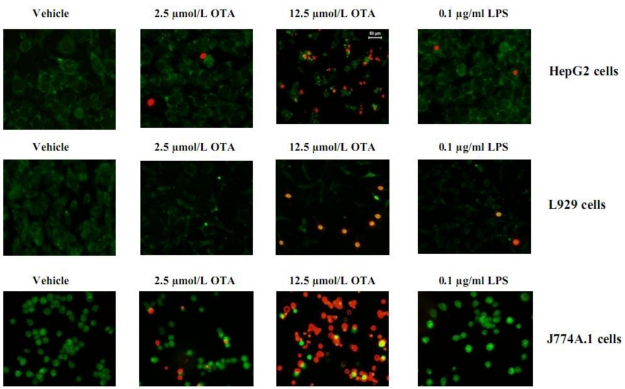
The cytotoxic effects of OTA and LPS on HepG2, L929 and J774A.1 cells were detected by LIVE/DEAD kit after exposure to the vehicle, 2.5, 12.5 µmol/L OTA or 0.1 µg/mL LPS up to 24 h. OTA and LPS were added 1 h after zero samples, (see Materials and Methods). The pictures were taken from at least five randomly selected square fields of microscope slides where we counted about 100 cells per field, The figure shows representative pictures for least three different individual experiments (Cytotoxicity of OTA and LPS).

Our previous studies have shown that OTA releases TNF-α from blood-free perfused rat livers in a dose- and time-dependent fashion without effects on liver vitality [7,9,13]. From these studies, we selected two OTA concentrations: 2.5 and 12.5 µmol/L. The cytotoxicity of OTA and LPS in this study was measured by MTT test (Table 1). Our results showed that 2.5 µmol/L OTA and 0.1 µg/mL LPS did not alter the survival of HepG2 cells, L929 cells, and J774A.1 cells in comparison with untreated cells (vehicle). The reduction of viability and proliferation in non-macrophages cells (HepG2, L929) was generally less than 5%, whereas 2.5 µmol/L OTA (but not 0.1 µg/mL LPS) reduced survival by about 15% in the mouse macrophage cell line J774A.1. In addition, for Kupffer cells and peritoneal macrophages, reduction was no more than 15% as measured by MTT (data not shown). The staining of L929, HepG2 and J774A.1 with the LIVE/DEAD kit confirmed the MTT data (Figure 1). With five-times higher concentration of OTA, significant toxicity of the tested cells was observed (Table 1, Figure 1). Our data indicates that the cells used in our study, with the exception of J774A.1, resist direct toxicity of 2.5 µmol/L OTA or 0.1 µg/mL LPS during 24 hours of exposure. Thus, these concentrations were used for further experiments.

**Table 1:** The cytotoxic effects of the vehicle, 2.5, and 12.5 µmol/L OTA and 0.1 µg/mL LPS after 24 h on J774A.1, HepG2, and L929 cell lines were detected by MTT test. OTA and LPS were added 1 h after zero samples (see Materials and Methods). Data are presentedas percent of control cells exposed to vehicle:absorbance of 24 h exposed cells/absorbance of 24 h control cells. Values represent the mean ± SEM of five cultures for each group, (* P < 0.05; ** P < 0.01; *** P < 0.001) (Effect of OTA and LPS on cell viability).

Percentages of cell viability after 24 h exposure to OTA or LPS (MTT)			
Cell type	HepG2 cells	L929 cells	J774A.1 cells
Treatments			
**Control (vehicle)**	100%	100%	100%
**2.5 µmol/L OTA**	<95% ± 5	<95% ± 5	85% ± 9 *
**12.5 µmol/L OTA**	75% ± 10 **	60% ± 10 ***	30% ± 20 ***
**0.1 µg/mL LPS**	<95% ± 5	<95% ± 5	<95% ± 5

### 2.2. TNF-α Release from Primary Macrophages and Macrophage-like Cells

In perfused rat livers, several types of cells may serve as a source of TNF-α, e.g., hepatocytes, sinusoidal endothelial cells, and Kupffer cells. In a previous study, OTA failed to induce TNF-α release in the blood-free perfused isolated rat liver when Kupffer cells were blocked *in vitro* by 15 µmol/L gadolinium chloride or when rats were pretreated *in vivo* with the Kupffer cell depleting clodronate liposomes [9]. Additionally, OTA-mediated TNF-α release originated from purified isolated rat Kupffer cells but not from rat hepatocytes or rat sinusoidal endothelial cells [9]. In order to extend our understanding for the cell sensitivity for OTA, we measured in this study OTA-mediated TNF-α release from additional cells (Table 2).

**Table 2:** TNF-α concentrations in the incubation medium of different cells at 24 h in the absence or presence of 2.5 µmol/L OTA or 0.1 µg/mL LPS. OTA and LPS were applied at 1 h from zero time (see Materials and Methods). Values represent the mean ± SEM of three cultures for each group, (* P < 0.05; ** P < 0.01; *** P < 0.001) (TNF-α concentrations in incubation the medium at 24 h).

Cell type	TNF-α concentrations in incubation medium at 24 h		
	Control (vehicle)	2.5 µmol/L OTA	0.1 µg/mL LPS
Kupffer cells	131 ± 10	1000 ± 40 ***	3000 ± 50 ***
Peritoneal macrophages	161 ± 10	1560 ± 90 ***	2600 ± 250 ***
J774A.1 cell line	111 ± 5	635 ± 45 ***	2115 ± 60 ***
Hepatocytes	47 ± 5	73 ± 5	776 ± 100 ***
HepG2 cell line	36 ± 2	48 ± 10	36
Sinusoidal endothelial cells	219 ± 10	235 ± 25	1925 ± 70 ***
L929 cell line	47 ± 10	53 ± 10	92 ± 5 ***

**Figure 2:**
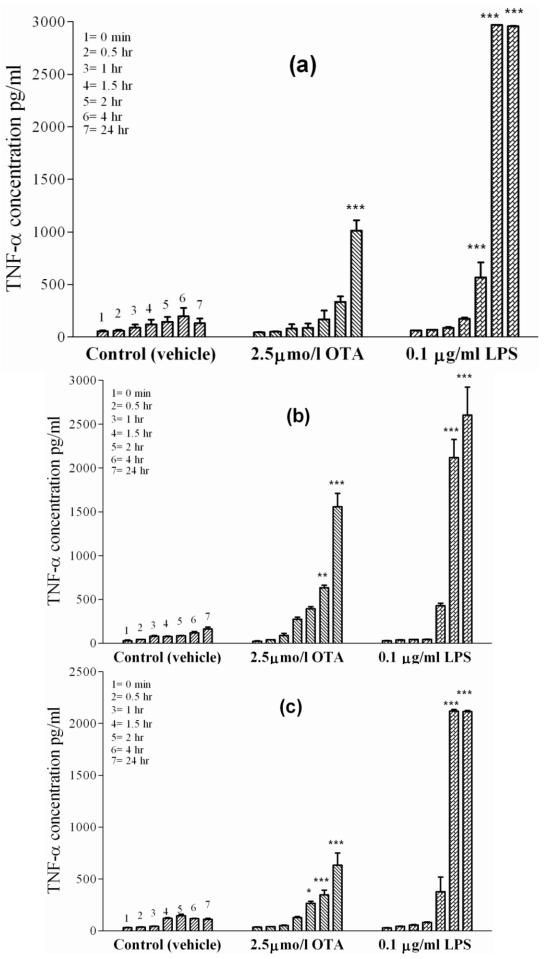
TNF-α concentrations were measured in incubation media of isolated cells in cell culture at 0, 0.5, 1, 1.5, 2, 4 and 24 h indicated by single columns 1–7, respectively. The cells were exposed to vehicle (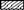), 2.5 µmol/L OTA (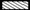) or 0.1 µg/mL LPS (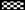). **Panel (a)** Kupffer cells. **Panel (b)** Peritoneal macrophages. **Panel (c)** J774A.1 cell line. OTA and LPS were added 1 h after zero samples (see Materials and Methods). Values represent the mean ± SEM of three cell preparations for each group (* P < 0.05; ** P < 0.01; *** P < 0.001) (TNF-α release from primary macrophages and macrophages like cells).

Primary rat macrophages, *i.e.*, Kupffer cells and peritoneal macrophages, were exposed to the vehicle or a standard dose of 2.5 µmol/L OTA or 0.1µg/mL LPS, respectively, during 24 h in culture. Kupffer cells (Figure 2(a)), which represent liver-placed resident macrophages, revealed basal TNF-α release in the absence of treatment (vehicle), which increased from 50 pg/mL at time zero to 131 ± 10 pg/mL after 24 h. However, 10-times as much TNF-α was released within 24 h when OTA was added. Onset of release began slowly during the first 4 h in the presence of OTA, but reached 1000 ± 40 pg/mL TNF-α (P < 0.001) at the end of the incubation period. Kupffer cells released TNF-α more rapidly and to a higher extent if exposed to 0.1 µg of LPS/mL. LPS caused significant (P < 0.001) release of TNF-α as early as 1 h after addition and reached 3000 ± 50 pg/mL after 24 h. Similarly, rat peritoneal macrophages (Figure 2(b)), representing non-residential freely movable macrophages, released basal TNF-α levels up to 161 ± 10 pg/mL in the absence of treatment (vehicle) after 24 h. An OTA concentration of 2.5 µmol/L stimulated these cells to release 1560 ± 90 pg TNF-α/mL after 24 h. The mycotoxin response on these cells was statistically significant (P < 0.01) at 4 h and became highly significant (P < 0.001) by the end of incubation (24 h), when TNF-α levels were 10-times higher than the basal TNF-α release. In response to 0.1 µg of LPS/mL, TNF-α was detected in the incubation medium after 1 h and became highly significant (P < 0.001) between 4 h and the end of incubation. After 24 h of LPS exposure, TNF-α concentrations reached 2600 ± 250 pg/mL.

In order to confirm the effects of OTA-mediated TNF-α release from rat macrophages, we investigated the effects of tested compounds on J774A.1, a mouse monocyte macrophage cell line. (Figure 2(c)) shows TNF-α release from these cells. The basal cell release was 32 pg/mL of TNF-α at time point zero and 111 ± 5 pg/mL of TNF-α after 24 h in culture treated with the vehicle. In the presence of OTA, TNF-α level significantly increased (P < 0.05) at 4 h, then became significant (P < 0.001) by the end point (635 ± 45 pg/mL). Much higher TNF-α level was reached in the presence of 0.1 µg of LPS/mL. Here, the TNF-α concentration was 2115 ± 60 pg/mL at the end of the 24 h incubation, showing that the effect of LPS on J774A.1 cells was much faster and three-times stronger than that promoted by OTA. Table 2 summarizes the results obtained and the significance for TNF-α release at the endpoint of 24 h in this set of experiments.

Other mycotoxins, *i.e.*, fumonisins [14,15] and aflatoxins [16], have also been found to interact with Kupffer cells disturbing the phagocytic properties. In these cases, it was reported that this interaction adds to the hepatotoxicity of the mycotoxins. Comparable with OTA, the fumonisins also forced a marked release of TNF-α from Kupffer cells [15]. Normally, antigen activation of Kupffer cells triggers vigorous activity for phagocytosis, during which they produce soluble mediators such as cytokines, prostanoids, oxygen radicals, and proteases [17]. While at low physiologic levels these mediators contribute to the physiological phagocytic process, their excessive production induces liver injuries [17,19]. We assume that mycotoxin-induced stimulation of Kupffer cells produces not only locally but even systemically circulating inflammatory mediators [20,22], as it occurs in response to gut-absorbed bacterial endotoxin LPS [22]. Furthermore, Kupffer-cell-derived mediators have been considered centrally responsible for switching and initiating several signaling cascades in hepatocytes, leading to apoptosis, but also to cell proliferation, and liver regeneration, especially in response to TNF-α [23,24]. Similar effects are expected under OTA exposure in murine livers where TNF-α mediated cell apoptosis was already reported [25,26].

Our observation of TNF-α release from macrophage cells by OTA contrasts with a report by Heller *et al.*, 2002. In their experiments on the human monocyte cell line THP-1, a dose-dependent reduction of LPS-induced TNF-α release occurred under increasing concentrations of OTA, likely due to the cytotoxicity of OTA. The authors treated the cells with 1 µg/mL LPS which is a 10-fold higher than the concentration used here, and also applied OTA together with LPS. Such differences in the experimental set-up and also in the method of detecting TNFα (which was done by a biological cytotoxicity test and not by ELISA) may have caused the discrepancies of OTA effects on monocytes.

### 2.3. TNF-α Release from Isolated Hepatocytes and HepG2 Cell Line

**Figure 3:**
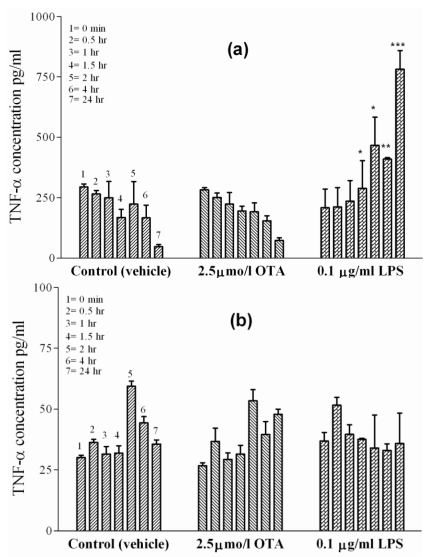
TNF-α concentrations were measured in incubation media of isolated cells in cell culture at 0, 0.5, 1, 1.5, 2, 4 and 24 h indicated by single columns 1–7, respectively. The cells were exposed to vehicle (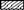), 2.5 µmol/L OTA (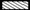) or 0.1 µg/mL LPS (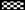). **Panel (a)** Hepatocytes. **Panel (b)** HepG2 cell line. OTA and LPS were added 1hr after zero samples, (see Materials and Methods section). Values represent the mean ± SEM of three cell preparations for each group (* P < 0.05; ** P < 0.01; *** P < 0.001) (TNF-α Release from Isolated Hepatocytes and HepG2 Cell Line).

Hepatocytes represent the major cell mass of the liver. To examine the possibility of hepatocyte participation in OTA-mediated TNF-α release from blood-free perfused rat liver [7,8], primary rat hepatocytes were prepared and kept in single-cell culture. The basal levels of TNF-α release from the hepatocyte cultures were higher in early cultures than those released from sinusoidal endothelial cells or Kupffer cells, but this level decreased with time from 295 ± 5 pg/mL at the starting point to 47 ± 5 pg/mL at 24 h in culture treated with vehicle (Figure 3(a)). An identical decrease of TNF-α occurred in the presence of 2.5 µmol/L OTA in hepatocyte cultures. This means that the blood-free perfused rat liver hepatocytes do not contribute to OTA-mediated TNF-α release. On the other hand, cytokine release was observed in the presence of 0.1 µg/mL LPS. Under LPS, the typical dropping of late TNF-α levels failed to occur and, instead, some slight increase was observed. This increase was significant at P < 0.05 at 1.5 and 2 h, became significant at P < 0.01 at 4 h, and then was highly significant at P < 0.001 after 24 h, at which time point it was three times above the initial TNF-α concentrations. Furthermore, in this study HepG2 cells (Figure 3(b)), an established *in vitro* model for human liver parenchymal cells, were used. TNF-α basal levels in the culture medium treated with vehicle increased only from 30 pg/mL to 36 pg/mL after 24 h. Neither 2.5 µmol/L OTA nor 0.1 µg/mL LPS altered the TNF-α concentrations in HepG2 cells when compared with untreated controls at any time point indicated. Thus, isolated hepatocytes and HepG2 cells failed to show any TNF-α release under OTA.

### 2.4. TNF-α Release from Isolated Sinusoidal Endothelial Cells and L929 Cell Line

**Figure 4:**
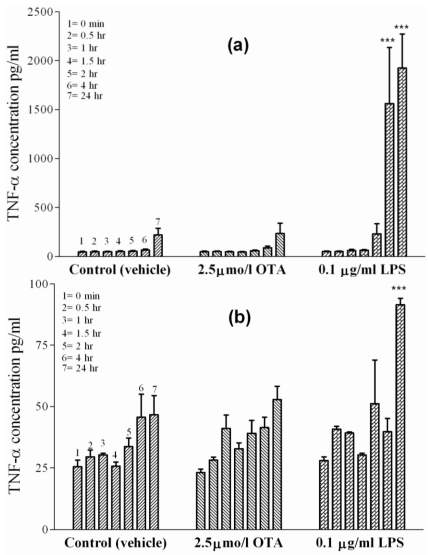
TNF-α concentrations were measured in incubation media of isolated cells in cell culture at 0, 0.5, 1, 1.5, 2, 4 and 24 h indicated by single columns 1–7, respectively. The cells were exposed to vehicle (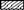), 2.5 µmol/L OTA (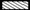) or 0.1 µg/mL LPS (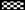). **Panel (a)** Sinusoidal endothelial cells. **Panel (b)** L929 cell line. OTA and LPS were added 1 h after zero samples were taken (see Materials and Methods). Values represent the mean ± SEM of three cell preparations for each group, (* P < 0.05; ** P < 0.01; *** P < 0.001) (TNF-α Release from Isolated Sinusoidal Endothelial Cells and L929 Cell Line).

Sinusoidal endothelial cells, together with Kupffer cells, line the liver sinusoids. The basal TNF-α release from separately prepared and cultured endothelial cells treated with vehicle (Figure 4(a)) was 45 pg/mL at time point zero and 220 ± 10 pg/mL after 24 h. In the presence of 2.5 µmol/L OTA, the TNF-α concentrations increased to identical levels, *i.e.*, from 49 pg/mL at time point zero to 230 ± 10 pg/mL after 24 h, indicating a lack of effect of OTA. In contrast, after adding 0.1 µg of LPS/mL into the incubation medium, a significant TNF-α release occurred as early as 4 h (P < 0.001) and reached 2000 ± 70 pg/mL after 24 h. 

We could not detect any significant release of TNF-α from rat liver sinusoidal endothelial cells exposed to OTA. TNF-α was, however, released from a human liver sinusoidal endothelial cell line during re-oxygenation injury following hypoxia. Here, the cytokine was released significantly in a time-dependent manner, while sinusoidal endothelial cell function decreased [28]. In addition, rat sinusoidal endothelial cells expressed TNF-α mRNA after i.v. injection of plasmid DNA attached to a cationic liposome complex (lipoplex) [29].

The connective-tissue mouse L929 cell line is a sensitive cell line for the apoptotic effects of TNF-α [30,31], indicating the presence of TNF-α receptors on the cells. These cells have been used as a biological marker to measure TNF-α-mediated cytotoxic effects by TNF-α inducers. In our experiments, the cells showed no TNF-α release upon exposure to vehicle or OTA (Figure 4(b)). In contrast, 0.1 µg/mL of LPS caused a slight release of TNF-α at 24 h, albeit much lower than in any other cell line. TNF-α concentrations were 91 ± 5 pg/mL under LPS after 24 h, which was significant at P < 0.001 in comparison to cells treated with vehicle (36 pg/mL).

From this data, we conclude that the release of TNF-α from non-macrophage cells such as hepatocytes, sinusoidal endothelial cells and L929 cells is in principle feasible but these cells did not respond to OTA under our cell culture conditions. However, cells released TNF-α upon incubation with LPS. A likely explanation for this discrepancy is to assume separate signaling mechanisms of OTA *versus* LPS on the different cell types.

### 2.5. Mechanism of OTA/LPS Mediated TNF-α Release: The Role of NF-κB

**Figure 5:**
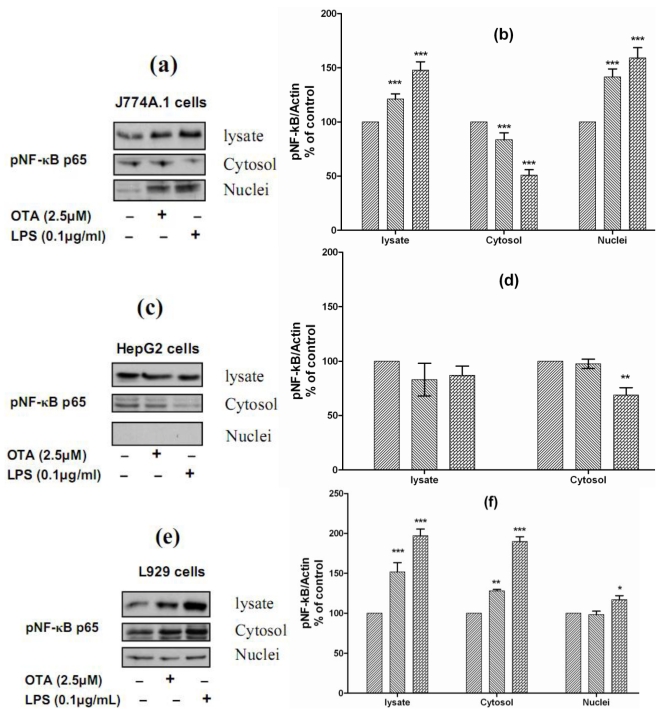
**Panels (a)**, **(c)**, and **(e)** Representative Western blots with antibodies detecting phospho-NF-κB p65 in the cell lysate, cytosolic and nuclear fractions after incubation with vehicle, 2.5 µmol/L OTA, or 0.1 µg/mL LPS up to 24 h. Panels **(b)**, **(d)**, and **(e)** Densitometric analysis of samples obtained from cells treated with vehicle (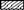), 2.5 µmol/L OTA (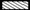) or 0.1 µg/mL LPS (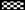). **Panel (a)** and **(b)** obtained from J774A.1 cells, **Panel (c)** and **(d)** from HepG2 cells, **Panels (e)** and **(f)** from L929 cells. Data obtained from three independent experiments, which presented as percent of control (mean ± SEM) after calibration for actin density. Marked columns were statistically different from controls (* P < 0.05; ** P < 0.01; *** P < 0.001)(The Role of NF-κB in OTA/ LPS mediated TNF-α release).

In all positive cells responding to both stimuli, the effects occurred more rapidly under LPS than under OTA. This also indicates that different signal cascades are involved for each of these two stimuli. Although far from being understood, earlier experiments have indicated that OTA-induced TNF-α release from the liver is likely to occur via the NFκB transcription factor pathway [7,9]. For understanding the mechanistic signals beyond OTA and LPS-mediated TNF-α release, we studied the ability of both stimuli to phosphorylate NF-κB p65 and the translocation from the cytoplasm to the nucleus. Therefore, we selected J774A.1 cells, which responded to both stimuli by releasing TNF-α, HepG2 cells, which did not respond to either of them, and L929 cells, which responded to LPS but not to OTA. We found that both stimuli phosphorylate and activate NF-κB p65 in J774A.1 cells and caused translocation from the cytoplasm to the nuclear compartment (Figure 5(a)). The densitometric analysis of Western blots showed that the levels of pNF-κB p65 in the cell lysate were increased significantly in response to OTA and LPS (121% ± 9 and 148% ± 15, respectively). The cytosolic levels of pNF-κB p65 were reduced significantly in response to both stimuli (83% ± 8 and 50% ± 10, respectively), this reduction resulted from translocation from the cytoplasm to the nucleus, due to the nuclear level of pNF-κB p65 increasing (142% ± 11 and 159% ± 15, respectively) in comparison with cells treated with the vehicle (Figure 5(b)). In contrast, both failed to activate NF-κB p65 in HepG2 cells. In these cells, the nuclear compartment was devoid of phosphorylated NF-κB p65. However, our data showed that LPS reduced the cytosolic level of pNF-κB p65 to 68% ± 10 of basal level (Figure 5c and Figure 5d). Interestingly, both stimuli could phosphorylate NF-κB in L929 cells, the levels detected in cell lysate were 151% ± 17 in response to OTA and 197% ± 13 in response to LPS in comparison with cells treated with the vehicle only. In these cells, LPS caused slight translocation to the nucleus, the cytosolic level was 190% ± 20, while the nuclear level was 117% ± 10 in comparison with vehicle (Figure 5e and Figure 5f). Obviously, TNF-α release in cells responsive to OTA or LPS was triggered via NF-κB, which was not activated in non-responsive cells. Our data also confirms findings of Ferrante *et al.*, 2008, showing that a low concentration of OTA (3 µM) induced NF-κB activation in J774A.1 cells starting from even 5 min after cell exposure [32]. 

The signaling of cells upon LPS stimulation is quite well understood. To date, pure LPS is known to induce TNF-α by Toll like receptor TLR4 [34]. TLRs make up a family of evolutionarily conserved recognition molecules, which are important signal transducers for the induction of mammalian innate immunity responses, including cytokine responses [33]. TLR4 requires co-receptors, such as CD14 and MD2. Altogether form a complex that activates TLR4 upon the binding at different adaptor proteins, including the myeloid differentiation factor 88 (MYD88) [35,36], Mac-1 [37], CXCR4, growth differentiation factor 5, and the heat shock proteins (HSP) 70 and 90 [38]. Then, downstream activation of protein kinases (e.g., IkB kinase and MAPKs) and transcription factors (e.g., NF-κB and AP-1) are induced, which ultimately lead to the expression of proinflammatory cytokine genes [39,40]. 

**Figure 6:**
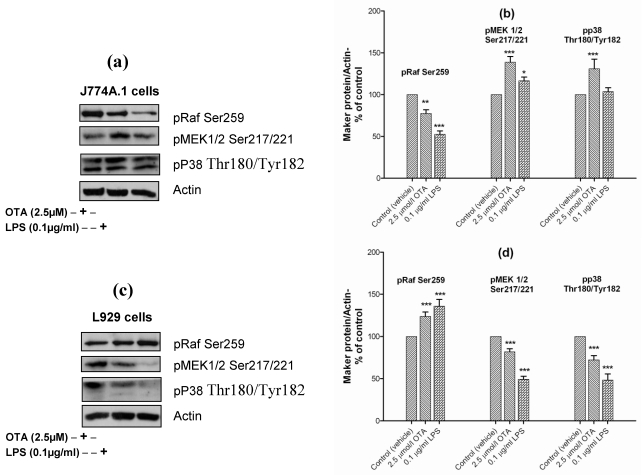
**Panels (a)** and **(c)** Representative Western blots with antibodies detecting phospho-Raf Ser259, phospho-MEK1/2 (Ser217/221), phospho-p38 (Thr180/Tyr182), and actin. Cells were incubated with vehicle, 2.5 µmol/L OTA, or 0.1 µg/mL LPS up to 24 h. Panels **(b)** and **(d)** Densitometric analysis of samples obtained from cells treated with vehicle (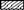), 2.5 µmol/L OTA (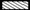) or 0.1 µg/mL LPS (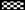). **Panel (a)** and **(b)** obtained from J774A.1 cells, while **Panel (c)** and **(d)** from L929 cells. Data obtained from three independent experiments, are presented as percent of control (mean ± SEM) after calibration for actin density. Marked columns were statistically different from controls (* P < 0.05; ** P < 0.01; *** P < 0.001)(OTA/ LPS mediated TNF-α release triggered via pRaf/MEK 1/2 and p38 pathways in J774A.1 cells).

OTA, on the other hand, activates mitogen-activated protein kinases (*i.e.*, extracellular signal-regulated kinase 1 and 2 (ERK1/2)), c-jun amino-terminal kinase (JNK), and extracellular-regulated protein kinase 38 (p38) in proximal tubular cells [41,42]. In our paper, we have examined the role of pRaf/MEK1/2 and p38 in OTA-mediated TNF-α release in comparison with LPS. In J774A.1 cells OTA and LPS reduced the level of the inactive form of Raf (pRaf Ser259) which increases the phosphorylation of MEK 1/2 at Ser217/221. However, only OTA was able to phosphorylate p38 at Thr180/Tyr182 (Figure 6(a)). Obviously, both pathways were used to activate NF-κB in mouse monocyte macrophage cell line in response to OTA (Figure 6(a)). The densitometric analysis of phosphorylated levels of NF-κB by OTA revealed lower levels than that induced by LPS. This corresponds to the lower TNF-α release compared with LPS. 

In contrast, in L929 cells OTA and LPS increased the level of pRaf Ser259, which suppresses MEK 1/2 Ser217/221 (Figure 6(b)) and, which occurred in parallel with p38 dephosphorylation (Figure 6(b)). It seems that the activation of NF-κB and TNF-α release from L929 cells response to LPS occurred without pRaf/MEK 1/2 or p38 activation. This provides evidence that LPS mediated TNF-α release occurs via several pathways at the same time. 

In monocyte macrophage cells, LPS uses membrane-bound CD14 [43,44]; whereas, soluble CD14 is retrieved in endothelial cells, as these cells lack the membrane bound CD14 form [44,45,46]. Thus, differential and cell-specific signaling has already been shown for LPS and could also explain why OTA only partially mimicked LPS. 

An alternative explanation for the lack of p38 occurrence in J774A.1 cells under LPS stimulation might be that the time point of our measurements at 24 h was too late for the activation kinetics of p38. However, because in all monocyte macrophage cells under study the toxins OTA and LPS continued to stimulate TNF-α release over the whole incubation time of 24 h we regard even the p38 signals at late time points confirmative. However, the effects on earlier time points including kinetic studies on signaling pathways will deepen the conclusions

Taken together, our data suggest differential and cell-specific signaling for TNF-α release, explaining why OTA only partially mimicked LPS. In this respect, OTA should trigger TNF-α release via the classical macrophagocytic signaling cascade, which involves TLR4, CD14, pRaf/MEK 1/2 or p38 and NF-κB. This would help to understand the observed activation of the pro-inflammatory response on distinct effector cells. Also, due to its different cell specificity, OTA may be a useful tool for studying selectively this type of TNF-α release mechanism.

## 3. Materials and Methods

### 3.1. Chemicals and Reagents

Ochratoxin A was purchased from CSIR Food Science and Technology, Pretoria, South Africa; it was free of lipopolysaccharide contamination as tested using the Limulus Amebocyte Lysate Endosafe^®^ KTA test kit from Charles River Wiga Sulzfeld, Germany. Lipopolysaccharide (*E. coli* serotype 0111:B4, product no. L-3012), collagen type VII from a rat tail, heat fetal calf serum, trypan blue, modified HANK's Ca^2+ ^free balanced salt solution and modified HANK's balanced salt solution with Ca^2+^ were purchased from Sigma-Aldrich Co. Steinheim, Germany. Collagenase type CIS 212 U/mg and RPMI 1640 medium were purchased from Biochrom AG, Berlin, Germany. HEPES was purchased from SERVA Electrophoresis GmbH, Heidelberg, Germany. Penicillin, streptomycin, trypsin/ EDTA, and Dulbeccos modified Eagles medium (DMEM) were purchased from GIBCO^TM^, Paisley, Scotland. DMSO and collagenase type NB 4 from *Cl. histolyticum* 0.161 PZU/mg was from SERVA Electrophoresis GmbH, Heidelberg, Germany. DNAase I was obtained from Boehringer-Mannheim GmbH, Mannheim, Germany, and Nycodenz^®^ 5-(*N*-2,3-dihydroxypropylactemido)-2,4,6-tri-iodo-*N*,*N*'-bis (2,3-dihydroxypropyl) isophthalamide was from AXIS-SHIELD PoCAS, Oslo, Norway. Pronase E was from E. Merck, Darmstadt, Germany. The enzyme-linked immunosorbant assay (ELISA) kit, Cytoscreen^®^, was purchased from BioSource International, Camarillo, Canada, with antibodies selective for detection of rat TNF-α., LIVE/DEAD® Reduced Biohazard Viability/Cytotoxicity Kit No.1. (L-7013) was purchased from Invitrogen Molecular Probes, Karlsruhe, Germany. Cell line L929 cells (NCTC clone 929, clone strain L, connective tissue, mouse), J774A.1 cells (mouse monocyte macrophage cell line), and HepG2 (human hepatoma cell line) were generously donated by Dr. Ayub Darji, Institute of Medical Microbiology, University of Giessen, Germany. 

### 3.2. Animals

Primary cells from the liver (Kupffer cells, sinusoidal endothelial cells, and hepatocytes) and peritoneal macrophages were isolated from male Wistar rats (200–280 g). The animals were fed *ad libitum* with Altromin^®^ standard diet and received water *ad libitum*. They were kept under 12 h light-dark cycles at 22 °C with ventilation. The health of the rats was routinely tested by sentinel animals, and the animals were found to be free of chronic infections and parasites.

### 3.3. Isolation of Sinusoidal Endothelial and Kupffer Cells

Kupffer cells and sinusoidal endothelial cells were isolated according to the collagenase/pronase digestion method previously described [47]. Briefly, blood-free isolated rat livers were perfused via the portal vein with a modified HANK's Ca^2+ ^free balanced salt solution at 7.5 mL/min for 5–10 min. Then livers were re-perfused with 80 mL RPMI 1.640 medium containing 34.4 mg/mL pronase E for 10 min at a flow rate of 10 mL/min. The medium was replaced by 100 mL RPMI 1640 medium containing 40 mg collagenase type CIS and 22.6 mg pronase E, and the livers were re-circulated for 30 min at 20 mL/ min with gassing by air containing 5% CO_2_. After that, the livers were dissected, and the tissues were digested for 15–30 min in 120 mL RPMI 1.640 medium containing 20 mg collagenase type CIS, 5.3 mg/mL pronase E, and 5 mg/mL DNase. During theincubation, the pH was carefully controlled to 7.4–7.6 by gassing with air containing 5% CO_2_ and by adding small amounts of 0.1 M NaOH. Subsequently, the cell suspension was filtered through sterile mesh 300 µm into three tubes and centrifuged (1.800 rpm, 10 min at 4 °C). The pellets were re-suspended in 10 mL HANK's balanced salt solution with Ca^2+^, and then centrifuged (1,800 rpm, 10 min at 4 °C). The cell pellets were re-suspended in up to 6 mL maximum of HANK's balanced salt solution with Ca^2+^; 14 mL of Nycodenz^®^ was also added to this suspension, which was then divided into two test-tubes. One mL of fresh HANK's balanced salt solution with Ca^2+^ was dropped carefully on the tube’s wall to form a clear layer above the suspension surface. The tubes were subjected to a density centrifugation at 3,500 rpm for 20 min. Afterwards, the upper three of four formed layers from each test-tube were collected, and the volume was replenished to 10 mL with cold RPMI 1640 medium, mixed, and injected into the JE-6B elutriation system and rotor (Beckman Instruments, Inc., Palo Alto, CA, USA). The speed was gradually raised from 19 to 27 mL/min. The outlets were collected in tubes, which contained pure endothelial sinusoidal cells. The outlets starting from 32 to 51 mL/min contained pure Kupffer cells. The cells were collected separately and centrifuged at 1800 rpm for 10 min at 4 °C. Finally, the pellets were re-suspended in the RPMI 1640 medium containing 10% heat-inactivated fetal calf serum and penicillin (100 IU/mL) and streptomycin (100 µg/mL). The cells were counted with a Bürker-Türk counter, adjusted to 2 × 10^6^ cells/mL, and seeded into tissue culture plates. Experiments were carried out under cell culture conditions.

### 3.4. Isolation of Hepatocytes

Hepatocytes were isolated by a collagenase perfusion method as previously described [48]. Briefly, isolated, blood-free, rat livers were placed in an experimental perfusion setup installed in a temperature-controlled hood. The livers were perfused with 75 mL of Ca^2+^ free Krebs-Henseleit solution containing 60 mg collagenase type NB 4 for 15 min in a temperature-controlled hood at 37 °C under 95% O_2_/5% CO_2_ gassing. After that, the livers were carefully dissected, and the tissue was digested for 5 min in the perfusate solutions by bubbling gas. The cell suspensions were filtered through double-layer sterile gauze into four test-tubes. The volume was adjusted by Ca^2+^-containing Krebs-Henseleit solution and centrifuged at 300–400 rpm (~50 grav) for 10 min. The pellets were re-suspended in Tyrode buffer containing 5.5 mM glucose, then washed three times, and filteredthrough four to six layers of gauze. The cells in suspension regenerated during 30 min in a shaker water bath adjusted to 37 °C and were gassed with 95% O_2_/5% CO_2_ gas. Finally, the cells were suspended in Dulbeccos Modified Eagles Medium (DMEM) containing 10% heat-inactivated fetal calf serum, penicillin (100 IU/mL), and streptomycin (100 µg/mL). The cells were counted under a microscope with a Bürker-Türk counter, adjusted to 2 × 10^6 ^ cells/mL, and then seeded in collagen pre-coated tissue culture plates. Experiments were carried out under cell culture conditions.

### 3.5. Peritoneal Rat Macrophage Preparation

Peritoneal macrophages were prepared as described previously [49] with some modifications. Rats received 5 µg concanavalin A in 1 mL buffer i.p. five days before being killed by carbon dioxide gas. The abdominal skin was removed, and 50–60 mL sterile phosphate buffer containing 50 IU heparin and 5% glucose was injected into the abdominal cavity. Five minutes later, about 50 mL of fluid was aspirated and transferred to sterile test-tubes. The test-tubes were centrifuged at 1200 rpm for about 10–15 min. Next, the supernatant was aspirated, and the cells were washed with phosphate buffer three times and then resuspended in DMEM supplemented with 10% fetal calf serum, 100 IU/mL penicillin, and 100 µg/mL streptomycin. The cell suspensions were seeded into culture plates and incubated at 37°C with a humidified 5% CO_2_ atmosphere for 2 h to allow the macrophages to adhere on the flask bottom. The culture medium with floating cells was discarded. Adhered cells were collected with trypsin and re-suspended in the culture medium. Then 5 mL of 2 × 10^6^ cells/mL were again seeded into culture plates and incubated at 37 °C with a humidified 5% CO_2_ atmosphere for 2 h to allow the purified macrophages to adhere on the flask bottom again. Experiments were carried out under cell culture conditions.

### 3.6. Cell Lines L929, HepG2, and J774A.1

Connective-tissue-derived mouse cell line (L929), human hepatoma cell line (HepG2), and mouse monocyte macrophage cell line (J774A.1) were cultured under routine conditions. Briefly, the cells were taken out from liquid nitrogen and thawed at 37 °C in a water bath. Next they were suspended in DMEM supplemented with 10% fetal calf serum, 100 IU/ ml penicillin, and 100 µg/mL streptomycin. The cells were seeded in tissue culture plates and incubated at 37 °C in a humidified 5% CO_2_ atmosphere. The cells were monitored during growth with replacement of the culture medium every 72 h until the plates became overcrowded with cells. Then, the cells were collected with trypsin, resuspended in culture medium, and transferred to several culture flasks. When these plates contained about 10 × 10^6 ^cells/plate, then 5 mL of new medium was added. Experiments were carried out under the cell culture conditions.

### 3.7. Cell Culture Conditions

Single cell preparations, each at 2 × 10^6^ cells/mL, were cultured at 37 °C in a humidifiedatmosphere of 5% CO_2_ in the air and incubated in 5 mL media supplemented with 10% heat-inactivated fetal calf serum and containing penicillin/streptomycin (100 IU and 100 µg/mL). Freshly prepared hepatocytes and sinusoidal endothelial cells were transferred to tissue culture plates pre-coated with collagen, while Kupffer cells, peritoneal macrophages, and cell lines L929, J774A.1, and HepG2 were seeded into uncoated tissue culture plates. Primary cells and cell lines were incubated without OTA or LPS during a pre-experimental period of 2 h. After that, at time point zero, medium samples were taken. One μg/mL (2.5μmol/L) OTA or 0.1 µg/mL of LPS were added to culture media 1 h after the zero-time sample. Whereas, the control cells or untreated were exposed to OTA vehicle.

### 3.8. Sampling Schedule

Culture medium samples were collected at 0, 0.5, 1, 1.5, 2, 4 and 24 h (end point). Samples were stored at −20 °C until they were analyzed by a rat TNF-α ELISA test system according to the company’s instruction.

### 3.9. Cytotoxicity Assay

Cytotoxicity of OTA and LPS was determined by 3-(4,5-dimethylthiazol-2-yl)-2,5-diphenyltetrazolium bromide (MTT) assay, detecting the cellular mitochondrial capacity to convert MTT tetrazolium salt to formazan. 300 µL of different cell suspensions (1 × 10^6^ cells/mL) was seeded in 24 well tissue culture plates coated with collagen type I. The cells pre-incubated 2 h prior to the addition of OTA at concentrations 0, 2.5 and 12.5 µmol/L or 0.1 µg/mL LPS into incubation media up to 24, 48, and 72 h. After that, the cells were incubated with the medium containing MTT for 4 hours. The cells were then lysed in DMSO. Finally, the absorbance was measured at 570 nm. 

### 3.10. Cell Viability

The applied concentrations of the toxins came from dose-finding experiments where concentrations of OTA between 0.5 and 75 µmol/L were selected; the concentrations used here were found not to significantly alter the viability of cells but effectively provoke TNF-alpha release. The viability of HepG2, L929 and J774A.1 cells were measured using LIVE/DEAD kit according to the company instructions. SYTO^®^ 10, a green fluorescent nucleic acid stain, is a highly membrane-permeant dye that labels all cells, including those with intact plasma membranes. DEAD Red (ethidium homodimer-2) is a cell-impermeant red fluorescent nucleic acid stain that labels only cells with compromised membranes. In brief, 300 µL of the cell suspensions (1 × 10^6^ cells/mL) were seeded on sterile glass cover slips, then cells were treated with; 0, 2.5 and 12.5 µmol/L OTA or 0.1 µg/mL LPS for 24 h. After that, cells were washed with HBSS and then the dye mixture of the LIVE/DEAD kit was added for 15 minutes at room temperature in the dark. Finally, cells were washed with HBSS again and were visualized under a fluorescence microscope. Fluorescence imaging was performed on a Leica DM IRE2 fluorescence microscope supplied with FITC filter (excitation 490 nm, emission 520 nm) and Texas Red filter (excitation 596 nm, emission 620 nm) from Leica, Wetzlar, Germany. The captured images were analyzed with the Leica Fluorescence Workstation software (FW4000). Live cells appeared fluorescent green and dead cells fluorescent red.

### 3.11. Cell Fractionation

Subcellular fractionation was performed as following: cells were washed with cold PBS, then 0.5 mL sucrose (0.25 M) containing a protease inhibitor cocktail was added for 5 min at 4 °C. After scraping, the content of plates was transferred into tubes. Cells were homogenized by a glass stick and their nuclei were pelleted by centrifugation (10 min, 3,500 rpm). The supernatant was used as cytosolic fraction, and nuclear pellets were re-suspended in IBP.7. containing protease inhibitor cocktail. The nuclear suspension was destructed by sonicating. The purity of fractionation was evaluated via detection of α-tubulin. 

### 3.12. Western Blotting

The cells were washed with phosphate-buffered saline and lysed in IPB.7 containing protease inhibitor cocktail (Catalog # P2714, Sigma-Aldrich, Germany). The protein was quantified by using Coomassie Plus^TM^ Protein Assay Reagent (Pierce, Täby, Sweden). The samples were subjected to SDS-PAGE. The separated proteins were transferred to a polyvinylidene difluoride membrane (Bio-Rad, Hercules, CA), and the protein bands were subsequently probed with antibodies.

Primary antibodies used in the analyses were phospho-NF-κB p65 (C22B4), phospho-MEK1/2 (Ser217/221), phospho-Raf (Ser-259) (Cell Signaling Technology); Actin (c-11): sc-1615 and phospho-p38 (Thr180/Tyr182)-R: sc-17852-R (Santa Cruz Biotechnology, Santa Cruz, CA). Secondary antibodies used in the analyses were goat anti-rabbit IgG sc-2004, goat anti-mouse IgG sc-2005 (Santa Cruz Biotechnology). The results were visualized by using the ECL or ECL plus detection kits (Amersham^TM^ GE Healthcare Bio-sciences AB, Uppsala, Sweden). Actin was used as a loading control. The results were analyzed with Image J version 1.34s software. 

### 3.13. Statistical Analysis

The data are presented as mean ± SEM for at least three separate trials for each experiment, obtained from different animals. A two-way ANOVA statistical test was used for analysis of variance, followed by a Bonferroni *t* test. *P* values of <0.05 (*), <0.01 (**), and <0.001 (***) compared with control values were considered statistically significant. Data was analyzed with Graphpad Prism 5.0 software (San Diego, CA, USA).

## 4. Conclusion

Our data indicate significantly different cell sensitivities to OTA *versus* LPS with respect to TNF-α release for the different cells and cell lines. Macrophages and macrophage-derived cell lines were most sensitive to both stimuli. Sinusoidal endothelial cells were less sensitive, and HepG2 cells and hepatocytes the the least sensitive. Inter-individual cell differences between OTA and LPS could be explained by different signaling pathways required for the stimuli of TNF-α release.
